# Increased risk of pneumonia amongst residents living near goat farms in different livestock-dense regions in the Netherlands

**DOI:** 10.1371/journal.pone.0286972

**Published:** 2023-07-05

**Authors:** Aniek Lotterman, Christos Baliatsas, Myrna M. T. de Rooij, Anke Huss, José Jacobs, Michel Dückers, Gert Jan Boender, Catherine McCarthy, Dick Heederik, Thomas J. Hagenaars, C. Joris Yzermans, Lidwien A. M. Smit

**Affiliations:** 1 Institute for Risk Assessment Sciences, Utrecht University, Utrecht, the Netherlands; 2 Netherlands Institute for Health Services Research, Utrecht, the Netherlands; 3 Wageningen Bioveterinary Research, Lelystad, the Netherlands; Universitair Kinderziekenhuis Koningin Fabiola: Hopital Universitaire des Enfants Reine Fabiola, BELGIUM

## Abstract

**Background:**

Previous studies, performed between 2009–2019, in the Netherlands observed an until now still unexplained increased risk for pneumonia among residents living close to goat farms. Since data were collected in the provinces Noord-Brabant and Limburg (NB-L), an area with relatively high air pollution levels and proximity to large industrial areas in Europe, the question remains whether the results are generalizable to other regions. In this study, a different region, covering the provinces Utrecht, Gelderland, and Overijssel (UGO) with a similar density of goat farms, was included to assess whether the association between goat farm proximity and pneumonia is consistently observed across the Netherlands.

**Methods:**

Data for this study were derived from the Electronic Health Records (EHR) of 21 rural general practices (GPs) in UGO, for 2014–2017. Multi-level analyses were used to compare annual pneumonia prevalence between UGO and data derived from rural reference practices (‘control area’). Random-effects meta-analysis (per GP practice) and kernel analyses were performed to study associations of pneumonia with the distance between goat farms and patients’ home addresses.

**Results:**

GP diagnoses of pneumonia occurred 40% more often in UGO compared to the control area. Meta-analysis showed an association at a distance of less than 500m (~70% more pneumonia compared to >500m) and 1000m (~20% more pneumonia compared to >1000m). The kernel-analysis for three of the four individual years showed an increased risk up to a distance of one or two kilometers (2–36% more pneumonia; 10–50 avoidable cases per 100,000 inhabitants per year).

**Conclusions:**

The positive association between living in the proximity of goat farms and pneumonia in UGO is similar to the previously found association in NB-L. Therefore, we concluded that the observed associations are relevant for regions with goat farms in the entire country.

## Introduction

In the recent past, an increasing body of research in The Netherlands and elsewhere has been devoted to studying the potential health risks of living in close proximity to intensive livestock farms. So far, multiple effects of exposure to livestock farming, and/or livestock related air pollution, have been reported [1–5]. These effects range from adverse effects such as an increased risk for zoonotic infections, decreased lung function and increased COPD exacerbations, to protective effects such as lower prevalence of certain immunological disorders e.g. asthma and allergies [1,2,4–13].

Previous epidemiological studies performed in the primary study area in the Netherlands, a livestock-dense area located in the southeast of the country, observed a currently unexplained increased risk for pneumonia among residents living close to goat farms between 2009–2019 [2,14–19]. Kalkowska et al. reported that the percentage of pneumonia cases attributable to proximity to goat farms (population attributable risk) was approximately 5% in the years 2009–2013. In a more recent analysis for the years 2014–2016, the population attributable risk for pneumonia of living within 2km of a goat farm was approximately 7% [20].

Q-fever, a zoonotic disease caused by *Coxiella burnetii*, is likely to explain the increased risk of pneumonia among neighboring residents of goat farms during and sometime after the epidemic that took place from 2007 until 2010. However, the Q-fever epidemic could not explain the increased risk of pneumonia in the period thereafter. As of 2011, Q-fever has been under control in both goats and humans as a result of preventive vaccination of goats and sheep and strict monitoring [21]. With reported incidence in humans having returned to pre-epidemic levels Q-fever is no longer a likely causative agent for increased pneumonia [22].

Given that the association of increased pneumonia incidence near goat farms is robust and consistent over an extended period of time in the southeastern region of the Netherlands, the question remains whether results are generalizable to other regions where people live close to goat farms [22]. This question was considered a key question that should be answered before the question of causality could be considered.

Therefore, in the present study, a new study region was included to assess whether the association between goat farm proximity and pneumonia was consistent across the Netherlands. Compared to the primary study area, this new study region has on average a lower background concentration of particulate matter air pollution but a similar density of goat farms. Results of this study will provide insights into potential influential regional differences regarding the prevalence of pneumonia among goat farms’ neighboring residents. This study is part of the Livestock Farming and the Health of Local Residents program (in Dutch: Veehouderij en Gezondheid Omwonenden—VGO).

## Materials and methods

### Study design and study population

An observational study was conducted in the Netherlands, based on longitudinal health data from electronic health records (EHR) in the Primary Care Database (PCD) of Netherlands Institute for Health Services Research (NIVEL) [23]. Every resident is obligated to be registered at just one practice, and general practitioners act as gatekeepers to secondary care. The EHR data used in this study comprised 68,000 patients on average per year (2014–2017), from 21 rural general practices (GPs) in the current study area located in the central and eastern part of the Netherlands. The study area included parts of the provinces of Utrecht, Gelderland and Overijssel (i.e. the current research area, hereafter referred to as UGO). The same procedure in collecting EHR data has been followed as described by previous research focusing on the province of Noord-Brabant and Limburg (i.e. the primary research area, hereafter referred to as NB-L) [2,16–18].

**Fig 1:**
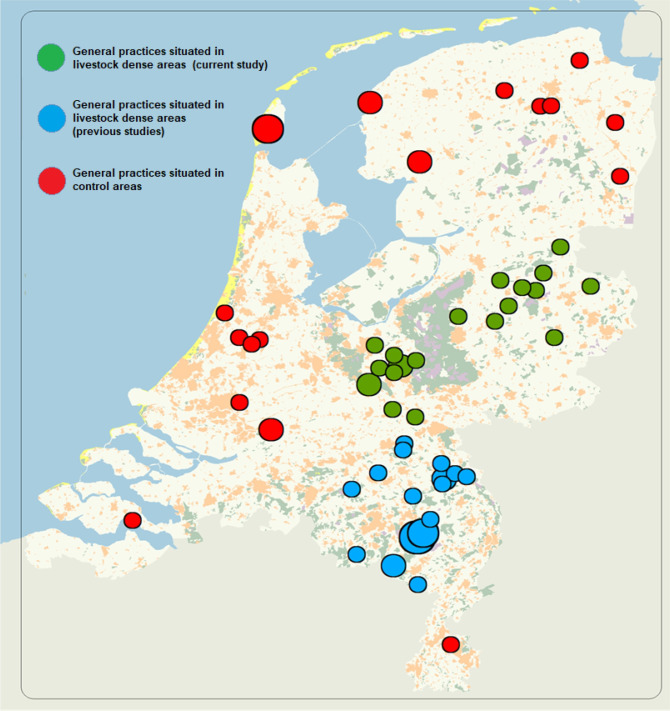
Overview of all participating GPs’ locations throughout the Netherlands, 2014–2017.

To enable comparisons between the current study area (UGO) and control areas, EHR data was derived from 66,000 patients per year from reference practices (the ‘control area’) located throughout the country (EHR of 22 GPs in rural areas with few intensive livestock farms). See Fig 1 for an overview of all participating GPs’ locations throughout the Netherlands. The recruitment of GPs to participate in the NB-L study has been described in detail by Kalkowska et al. 2018 [20].

In primary care in the Netherlands diagnosed health problems are registered based on the International Classification of Primary Care (ICPC) (Pneumonia is code R81) [24]. Annual prevalence of pneumonia was based on “care episodes”; every care episode includes all patient encounters for the same health problem, within an ICPC code. In addition, the yearly prevalence of herpes zoster (ICPC code S70) has been included as a control diagnosis, considering the lack of a theoretically expected association with exposure to farming.

For studying associations between residential distance to a goat farm and pneumonia diagnoses, patients registered at all 21 GP’s in UGO were included. Because the study focused on environmental exposure to livestock farms, patients living at a livestock farm were excluded from these analyses (because of the potential of occupational exposure). Patients were also excluded when they were registered at more than one residential address.

### Ethical approval

The NIVEL Primary Care Database (PCD) complies with the regulations of the Dutch Data Protection Authority and the Dutch law regarding use of health data for epidemiological research (Dutch Civil Law, Article 7:458). Medical information and address records were kept separated with the support of a Trusted Third Party (“Stichting Informatie Voorziening Zorg: IVZ”, Houten, The Netherlands).

### Exposure assessment

The presence of livestock farms in UGO has been determined using “Bestand Agrarische Bedrijfssituatie (BAB)”. Farm-level data from 2016 were used. BAB is a national inventory, compiled by the Netherlands Enterprise Agency (Dutch acronym: RVO). This database provides information on animal species and number, geographic coordinates of farms and associated emissions per year. All patients’ home addresses were geo-coded based on Dutch cadastral data, and ArcGisPro (version 2.5) and R (version 3.6.1) was used to determine the shortest distance between residences and farms. A goat farm was defined as a location where a minimum of 50 goats were being kept.

### Data analyses

This study evaluates associations between pneumonia and the proximity of goat farming by using complementary data analysis approaches: i) Multi-level analysis taking into account the hierarchical structure of the data (registered patients nested in GP) to compare the annual prevalence of pneumonia between UGO and the control area; ii) random-effects meta-analysis to study associations between pneumonia and pre-defined distances between goat farms and patients’ home addresses for each GP practice and summarizing associations, and iii) a spatial kernel analysis that assumes a hazard of developing pneumonia that is dependent on the distance of the residence of each patient to every individual goat farm.

#### Multi-level analysis

The prevalence of pneumonia between 2014–2017 is compared between study areas using (multi-level) logistic regression analyses, adjusting for age, sex, the number of days a patient was registered with the GP, and clustering of patients in GP’s [25]. Four-year prevalence, yearly prevalence for 2014–2017 and Odds ratio’s (OR) and 95% confidence intervals (95%CI) were calculated. Two-sided testing was conducted considering the interest in differences between study areas.

#### Random effects meta-analysis

Associations between presence of a goat farm within a radius of 500m, 1000m and 2000m from the residential address and pneumonia were determined using logistic regression, and expressed as OR and 95%CI. People with one or more pneumonia episode(s) over the years 2014–2016 were compared to people who had no pneumonia in these years. For a comparison of UGO and NB-L we restricted the analysis to 2014–2016. The year 2017 has been left out of this specific analysis, because there was no complete dataset available for NB-L for 2017. All associations have been adjusted for age, sex and the presence of a poultry farm within 2000m. The latter adjustment was considered because earlier publications showed an increased risk of pneumonia for people living in the proximity (up to 2000m) of poultry farms [14,20]. The associations were first calculated for all individual GPs after which results were combined in a random-effects meta-analysis. GPs were excluded from the meta-analysis when there were too few registered patients living within a certain radius of a goat farm to calculate a GP-specific association. In the meta-analysis combined effect estimates were calculated for 1) all GP’s in UGO, 2) all GP’s in NB-L (for comparison, these previously estimated effects were also displayed) [25], and 3) all GP’s of both study areas combined. I^2^ was calculated as a measure of heterogeneity in associations between GPs or research areas underlying the meta-analysis effect-estimates. The size of the study population is somewhat smaller in UGO compared to NB-L therefor some results might not prove statistically significant, but are given to evaluate similarity in outcomes with NB-L.

#### Spatial kernel model

Associations between pneumonia and residential distance to goat farms (as well as to other types of farms) in UGO were determined using kernel-analysis [17,20,22]. The kernel model calculates the average added risk of pneumonia per goat farm within a certain radius of the patient’s home address. The kernel hazard function assumes that the potential additional pneumonia hazard is equal for all individuals living within a particular distance/radius *d* from a livestock farm of a specific type and 0 for all individuals living further away. In addition, the model includes a spatially uniform background pneumonia hazard (independent of residential distance to any type of livestock farm). In the univariate analyses the radius d was varied between 500 and 2000 m with steps of 500 m. For further technical details we refer to Kalkowska et al. [20]. The combined univariate and multivariate analyses are also used to calculate the population-attributable Risk (PAR), defined as the percentage of pneumonia cases that could be, or would be, prevented if no one was living within that radius of a goat farm, when you assume a causal relationship [6,20,22]. In the calculation of the PAR we used the estimated background pneumonia hazard to estimate the percentage of pneumonia cases that would remain if no one was living within the given distance(s) of farms.

#### Splines

Associations between pneumonia or herpes zoster (control diagnosis) and distance to a farm were visualized using spline plots according to Post et al. [22]. This allows comparisons of the shape of these associations.

#### Effect of weighing the number of goats

Finally, the effect of farm size (i.e. number of animals) on top of presence of a goat farm (yes/ no) was explored as previously described by Huijskens et al. [26]. The OR (95%CI) for an interquartile range increase in the number of goats were calculated. Additionally, the number of goats present in the different radii of peoples’ homes was assessed as a categorical variable (e.g. no goats, ≤ median number of goats, > median number of goats).

#### Sensitivity analyses

We adjusted for several additional potential confounding factors that could influence the association between goat farming and pneumonia; the presence of other farm types, residential concentrations of ambient NO_2_ (as a proxy for annual average concentrations of traffic related air pollution at the residential address, determined with previously developed and validated models from the EU ELAPSE project [27], and age as a squared term (because of the non-linear association between age and pneumonia).

Analyses were performed in R version 4.0.3 in SAS, version 9.4 (SAS Institute, Inc., Cary, North Carolina, USA, 2016) and in STATA versions 12.1 and 14.0 (StataCorp LP, College Station, TX, USA). The kernel-analysis was programmed in Wolfram Mathematica v.10.4 and executed in v11.3 (Wolfram Research, Inc., Mathematica, Version 11.3, Champaign, IL, 2018). The program-code for the kernel-analysis has been previously published [20]. Statistical significance was set at p < 0.05.

## Results

### Descriptive of study population

In total, 21 GPs were included in UGO, with 68.000 patients on average per year. For the control area 22 GPs were included with 66,000 patients on average per year (Table 1). The two groups were comparable for age and sex, although the proportion of children was somewhat lower in the control area. The national prevalence of pneumonia in primary care, including all urban and rural residents, was 1.63% per year in 2017 and 1.61% in 2019 [23,28].

**Table 1:** Comparing UGO and control area for pneumonia prevalence and differences in risk per year.

	2014	2015	2016	2017
NO. PATIENTS IN UGO (N PRACTICES)	58,291 (20)	68,698 (21)	71,396 (21)	74,093 (21)
PREVALENCE (%) OF PNEUMONIA	1.61	1.81	1.64	1.92
NO. PATIENTS IN CONTROL AREA (N PRACTICES)	72,469 (22)	71,908 (22)	69,806 (21)	50,139 (18)
PREVALENCE (%) OF PNEUMONIA	1.27	1.43	1.37	1.41
OR (95%CI) PNEUMONIA	1.41 (1.02–1.95)***	1.44 (0.99–2.09)**	1.38 (0.98–1.95)***	1.40 (1.01–1.93)**
OR (95%CI) HERPES ZOSTER	1.05(0.90–1.22)*	0.93(0.78–1.12)*	0.97(0.80–1.17)*	1.12(0.92–1.36)*

**UGO (2014; 2017):** Percentage women: 49.8%; 49.8%. Average age: 41.3 (SD 23.9); 41.3 (SD 24.2). Percentage children (0–14 year): 18.3%; 18.5%. Percentage elderly (65+): 19.8%; 20.4%.

**Control area (2014; 2017)**: Percentage women: 49.7%; 49.4%. Average age: 41.9 (SD 23.5); 42.0 (SD 23.5). Percentage children (0–14 year): 16.6%; 15.9%. Percentage elderly (65+): 19.8%; 20.0%.

* not significant.

** p<0.10.

*** p<0.05.

In total 65,251 patients were included for analysis (50,696 adults (>18 years of age in 2016) and 14,555 children), see Table 2. Depending on the availability of address information not all patients could be included. For studying the associations between residential distance to a goat farm and pneumonia 2,905 patients were excluded because they lived at a livestock farm and nine patients were excluded because they were registered at more than one residential address. Over the period of 2014–2017, 2,591 patients were diagnosed with pneumonia leading to a four-year-prevalence of 4.0%; 4.2% among adults and 3.1% among children. Table 2 also shows that approximately 30% of patients live within 2000m of one or more goat farms (information on other types of livestock farming are shown in S1 Table in S1 File).

**Table 2:** Demographic characteristics of research population in the provinces of Utrecht, Gelderland and Overijssel.

CHARACTERISTIC	UGO NUMBER (%)
**TOTAL POPULATION**	65,251 (100)
POPULATION IN 2014	50,577 (78)
POPULATION IN 2015	59,377 (91)
POPULATION IN 2016	61,577 (94)
POPULATION IN 2017	64,041 (98)
BORN BETWEEN 2014 AND 2017	2,287 (3.5)
BORN BETWEEN 2014 AND 2016	1,913 (2.9)
MEN	32,696 (50.1)
WOMEN	32,555 (49.9)
AGE CATEGORY*	
< = 19	14,555 (22.3)
19< = < 37	13,100 (20.1)
37< = < 52	11,945 (18.3)
52< = < 66	12,679 (19.4)
66< = < 106	12,972 (19.9)
URBANIZATION	
1 (MOST URBAN)	450 (0.7)
2	4,202 (6.4)
3	4,028 (6.2)
4	29,289 (44.9)
5 (LEAST URBAN)	27,282 (41.8)
MINIMUM OF 4 YEAR ** REGISTRATION AT THIS GP	38,524 (59.0)
GP PATIENT IN GELDERLAND	31,777 (48.7)
GP PATIENT IN OVERIJSSEL	29,427 (45.1)
GP PATIENT IN UTRECHT	4,047 (6.2)
AGE (MEAN, SD)*	42.11 (24.03)
YEAR OF BIRTH (RANGE)	1911–2017
PERCENTAGE LOW INCOME (N = 64.670 AV, STD; RANGE)	40.67 (4.19; 17–69)
GOAT FARMS WITHIN *X*M RADIUS OF HOME	
>2000m	46,356 (71.0)
1000-2000M	15,575 (23.9)
500-1000M	2,796 (4.3)
<500M	524 (0.8)

* age in 2017 for UGO.

#### Comparing UGO to control area

Pneumonia was diagnosed roughly 40% more often in UGO compared to the control region. The differences in prevalence between UGO and the control area for four consecutive years are listed in Table 1. The differences in prevalence are adjusted for age, sex, and duration of registration at GP. Differences in risk of pneumonia for UGO and the control area were statistically significant and the OR’s range from 1.38 to 1.44. For the control diagnosis herpes zoster no statistically significant differences were detected between UGO and the control area.

### Meta-analysis residential distance to goat farms

**Fig 2:**
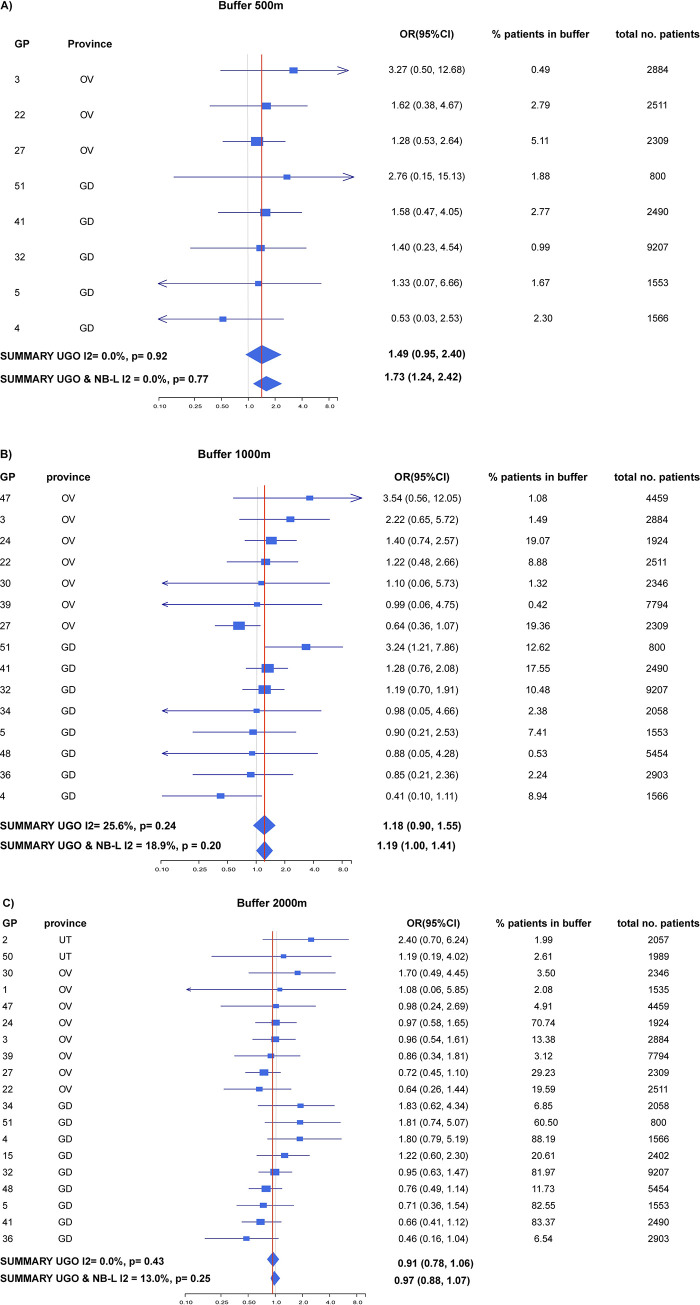
Results of the meta-analysis of the logistic regression expressed in odds ratios for individual GPs in Utrecht, Gelderland and Overijssel and the overall odds ratio. The associations between the presence of a goat farm within a radius of **A)** 500m, **B)** 1000m and **C)** 2000m from the home address and pneumonia are shown for the whole study population for the years 2014–2016 (OR (95%CI)). All associations have been adjusted for age, sex and the presence of a poultry farm within 2000m. The window-shape reflects the 95% CI from the combined effect estimations. The size of the square-shapes around the individual effect estimations reflects to which extent this contributes to the combined effect and is dependent on the accuracy of the estimation.

Meta-analysis showed an association at a distance of less than 500m (~70% more pneumonia compared to >500m) and 1000m (~20% more pneumonia compared to >1000m), with no evidence for heterogeneity between GPs or study areas (overall I^2^<20%) (Fig 2). For other farm types the results from the meta-analysis did not show any consistent associations with pneumonia (S2 and S3 Tables in S1 File).

See for the meta-analysis for NB-L Post et al. in 2019 [22].

### Kernel analysis

The multivariate kernel analysis for 2014, 2016 and 2017 showed an increased risk of pneumonia up to a residential distance to goat farms of 2 km, 1 km and 1.5 km respectively. For 2015 no statistically significant distance-dependent association was found (i.e. add pneumonia risk was not significantly different from zero for any of the distance radii considered in the analysis). A 1.9–36.1% increase in pneumonia prevalence, resulting in 10 to 50 avoidable cases per 100,000 inhabitants per year was seen among residents living in the proximity of a goat farm. Results of the kernel analysis are shown in the (S4 Table in S1 File). No statistically significant risk increase in relation to residential distance to poultry farms was found. However, for all four consecutive years the kernel analysis, in contrast to the other methods used in this study, also detected significant pneumonia risk increases for residents in the vicinity of sheep farms. For the control condition herpes zoster, no increased risk was found in the vicinity of any of the farm types for any of the years.

### Spline plots

**Fig 3:**
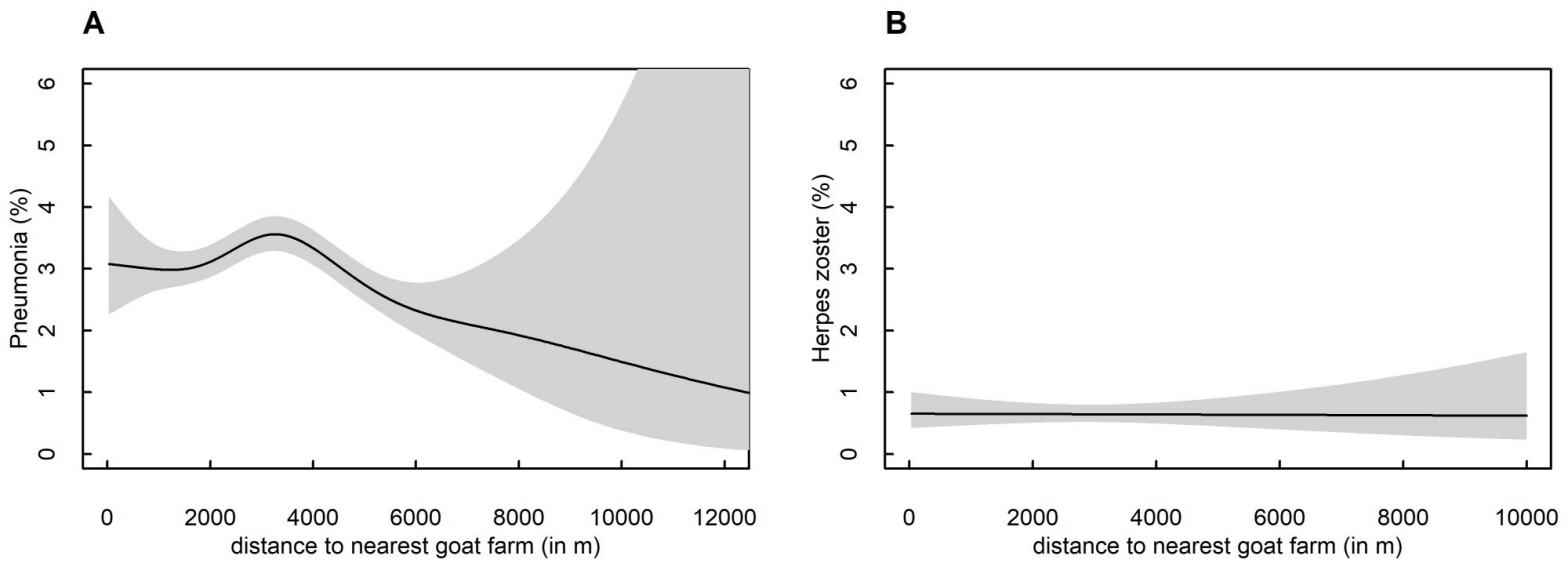
Spline plots showing the associations between residential distance to nearest goat farm and being diagnosed with (A) pneumonia or (B) herpes zoster in the period 2014–2017. **A** Association between residential distance to nearest goat farm and being diagnosed with pneumonia in the period 2014–2017 with a spline for all ages in the provinces of Utrecht, Gelderland and Overijssel (n = 65,251; adjusted for age, sex, presence of pig farm within 500m, a sheep- or mink farm within 1000m and presence of a poultry farm within a 2000m radius. The p-values of the spline is <0.001, while a linear model shows a p-value of 0.06. The spline plot shows an increased prevalence of 3–4%, after which the prevalence decreases to 1–2%. B Association between residential distance to nearest goat farm and being diagnosed with the control diagnosis herpes zoster in the period 2014–2017 with a spline for all ages in the provinces of Utrecht, Gelderland and Overijssel (n = 65,251; adjusted for age, sex, presence of pig farm within 500m, a sheep- or mink farm within 1000m and presence of a poultry farm within a2000m radius.

Spline plots were created to compare the shapes of associations between residential distance to nearest goat farm and with pneumonia diagnosis or control diagnosis (herpes zoster) (Fig 3). Plots show that pneumonia prevalence tends to decline with increasing distance to nearest goat farm. In contrast, herpes zoster diagnosis prevalence was not related to distance.

### Sensitivity analysis

The association between distance to goat farms and pneumonia was not influenced by other possible relevant sources within the surroundings (traffic-related air pollution and living in the proximity of other types of livestock farms than goats or poultry), nor by the functions of age (age*age). The influence of these additional corrections has been assessed by meta-analysis (S2 Table in S1 File).

Fixed effect modelling was performed as a sensitivity analysis, the same results were obtained as with random effect modelling. Goat density around the home address did not show a significant effect on the risk of pneumonia on top of presence of a goat farm (S5 Table in S1 File), also not when analyzed as a categorical variable (no goats, equal or less than median number of goats, more than median number of goats (S6 Table in S1 File).

## Discussion and conclusions

This study shows a statistically significant positive association between living in the proximity of goat farms and pneumonia. This is in line with earlier findings in a different region. Several complementary methods of analysis have been applied. They all supported the association of goat farming and pneumonia. This study highlights the previously identified epidemiological association between goat farming and increased pneumonia risk to also be apparent for other livestock-dense areas in the Netherlands.

### Main findings

In UGO, on average 40% more pneumonia cases are diagnosed annually compared to the control area. In NB-L, 50–60% more pneumonia cases are diagnosed, as previously published by Baliatsas et al. in 2020 [25], showing an average prevalence of about 2% in the primary study area for the period 2014–2016. This is slightly higher than the average prevalence observed in UGO, which was around 1.7%. Associations between residential distance to goat farms and the occurrence of pneumonia found in UGO are in line with associations identified in NB-L.

The consistency of the associations is shown through similar findings for comparable study areas. Both the study and control groups were selected on the basis of the same criteria as in previous VGO-studies and both of them consisted of similar areas in terms of urbanicity (rural areas). The positive association between living in the proximity of goat farms and pneumonia in UGO is similar to the previously found association in NB-L and therefor strengthens the conclusions from previous VGO-studies.

The presence of goat farming in the vicinity of communities cannot be the only explanation of the relatively large increase of pneumonia diagnoses in livestock dense rural areas. Other risk factors such as regional air pollution by fine dust (primary particulate matter and secondary inorganic aerosols) and endotoxin emission from other livestock farms may play a role as well. The increased prevalence of pneumonia in UGO confirms the previously found increased prevalence in a livestock dense region. Slight differences in the prevalence of pneumonia between the study areas versus the control area could be caused by livestock farming but also by other unknown factors. Since the UGO area was selected based on similar density of goat farms and population, but less industry present, the results support a causal association with goat farm exposures.

As a result of the Q-fever outbreak people could have developed a more negative attitude towards livestock farming and an increased alertness for respiratory symptoms. However, the epicenter of the Q-fever outbreak was located in NB-L and the number of cases in UGO was limited [29]. There has been debate whether attitude towards livestock farming could influence people’s care seeking behavior. It was previously found that, in NB-L, residential exposure to livestock was associated with fewer GP visits and self-reported symptoms for respiratory and other conditions [29]. Effects of attitude towards livestock farming on pneumonia for the NB-L GP patient population led to nondifferential misclassification of self-reported pneumonia with regard to participants’ attitude. No indication was found that the association between proximity to goat farms and pneumonia was modified by attitude [30].

Our interpretation that goat farming is associated with an increased pneumonia risk is strengthened by the observations that herpes zoster, considered as a control diagnosis, was not clearly associated with goat farming: the prevalence appeared not to be different between study areas and the control area, and also no association with distances to goat farms or other livestock farms was found.

### Strengths

GP diagnoses were used in this study, based on a large and reliable health care database [23]. Advantages to this approach are that a sufficiently large sample size from the general public is obtained relatively easily, which is not biased by selection since the whole GP-registered population is included in the analyses and each Dutch inhabitant is registered with only one GP in the same residential area. Moreover all GP’s in the Netherlands register pneumonia on the basis of the same classification system and diagnostic code. The quality of registration has been checked and approved by a previously set standard [18]. However, misclassification, to some extent, of the diagnosis pneumonia cannot be ruled out. Pneumonia is an infection of the lower respiratory tract with inflammation of the alveoli and surrounding lung tissue. GP diagnosis of pneumonia is mostly based on clinical presentation only [23], since in the case of a bacterial pneumonia there is a need for early antibiotic treatment and waiting for the results of a thoracic X-ray (the golden standard for definitive diagnosis) takes too long. Therefore, in the Netherlands, for uncomplicated cases of pneumonia confirmation by X-ray is not common practice. Among participating GPs in both NB-L and UGO it was explored whether physicians would unambiguously distinguish between pneumonia and acute bronchitis [31]. Providing sufficient information, pneumonia was recognized as such by almost all participating GPs, while the distinction with acute bronchitis seemed more difficult when provided with less information or with an ambiguous C-reactive Protein (CRP) test result. Distinguishing bacterial from viral pneumonia is not always clear cut and the causative agent remains almost always unknown. These limitations, however, weigh the same for all participating GP’s in the research areas and the control area.

For analyzing associations between residential distance to goat farms and pneumonia, a random-effects meta-analysis was performed in which associations per GP are analyzed and subsequently combined. Benefits of this method are that results per GP are presented and can thus be interpreted, and absolute differences in prevalence of pneumonia between GP’s have probably less impact on the found associations compared to analyzing all GPs together. A limitation of this method is that GPs with relatively few goat farms in their proximity cannot be included, because this limits the power to detect associations for those GPs, resulting in an overall reduction of statistical power.

Therefore, in addition to the meta-analysis, a kernel analysis was used. In the kernel-analysis the full study population is included. The advantage of this method is that the residential distance to each individual livestock farm is included to model the probability of pneumonia, with no pre-defined distances, while the meta-analysis looks at a number of pre-set buffers (i.e. minimum of one goat farm within a 500m, 1000m or 2000m radius). A limitation of the kernel-analysis is that the method requires considerable computing power. Overall, both methods showed similar findings, except when zooming in on the distances defined at risk. But this was not unexpected considering the higher level of precision that can be acquired with kernel-analyses.

There is not one preferred method for these analyses. Comparable results between the analysis methods strengthen the separate analyses’ results and show the results are robust for the underlying assumptions of the used models. Inconsistencies in results can occur due to a lack of distinguishing abilities, choice in cut-off points for distances or the presence of multiple livestock farms within a certain distance.

### Limitations

Usage of GP data is limited by the amount of information available regarding possible confounding variables. Although the demography of UGO and control area are similar for main characteristics such as age and sex. Previous research has shown that the influence of variables such as smoking habits and socio-economic status on the associations between pneumonia (among other outcomes) and residential distance to livestock farms (including goat farms) is very limited [8,14,15]. Additional analysis showed that the association between residential distance to goat farms and pneumonia is not influenced by other possibly relevant local sources of air pollution in UGO, specifically traffic related air pollution and living in the proximity of other farms.

An additional source of uncertainty in the risk estimations is the exact location and size of the farms. Any deviations in animal numbers from the BAB-data would result in non-differential misclassification of the exposure, leading to underestimation of the true risk for pneumonia among neighboring residents of goat farms [32].

### Future research

More insight into the mechanisms behind these observed epidemiological associations is needed, in particular the cause of the excess risk needs to be identified. New research has already been initiated and focuses on discovering what is causing these pneumonia cases related to residential goat farm exposure [33]. This research includes patients with residential goat farm exposure related pneumonia and compares them with a) patients with pneumonia without residential goat farm exposure, b) healthy people with and without residential goat farm exposure.

Also the effect of specific goat farm characteristics and goat farm management information is considered to help explain the association. In our study there was no added effect measurable of farm size (i.e. number of goats). This might be due to a lack of power. The goat farming industry is a relatively small livestock farming sector within the Netherlands compared to other types of livestock farming (e.g. dairy cows, pig or poultry). Other explanations may be differences between larger and smaller farms in for example farm management, herd health, type of stable or compost management.

Possible temporal effects on the prevalence of pneumonia should also be further assessed. A recent study showed no clear effect of season on the odds ratio of pneumonia amongst neighboring residents of goat farms. Although pneumonia prevalence varies throughout the year, the association between goat farm exposure and pneumonia seems to be not season-specific [19]. Specific recurring events on goat farms, such as cleaning out the stables, handling compost or large animal movements, remain understudied and are analyzed as possible determinants in ongoing research.

Further findings that require more detailed research to fully understand are the associations, previously found, between pneumonia and poultry farms and, from this study (in the kernel analysis), sheep farms. In previous studies the increased risk of pneumonia for people living close to poultry farms was limited compared to goat farms and fluctuated over the years [18]. Possible health effects remain a topic of interest in regions with a high density of poultry farms, due to the relative high rates of particulate matter emission [34].

## Conclusions

In the current study, we established that the increased risk for pneumonia when living in close proximity to goat farms is consistent, not only throughout eleven consecutive years (2009–2019), but also across different regions in the Netherlands. Therefore, we assume that the observed associations are relevant for regions within the country. There seems to be an unknown factor related to goat farming, that is associated with the risk of pneumonia. The findings described previously and in our current analysis have led to a moratorium for new goat farms and limited possibilities to expand for existing goat farms across the country. However, there is still no clear lead on what is causing this increased risk for pneumonia in relation to residential exposure to goat farming. Follow up studies will address underlying causal factors or mechanisms are involved in the increased risk for pneumonia among people living in close proximity to goat farms.

## Supporting information

S1 File VGO-UGO supplements. (DOCX)
