# Correction: Increased risk of pneumonia amongst residents living near goat farms in different livestock-dense regions in the Netherlands

**DOI:** 10.1371/journal.pone.0355773

**Published:** 2026-08-11

**Authors:** Aniek Lotterman, Christos Baliatsas, Myrna M. T. de Rooij, Anke Huss, José Jacobs, Michel Dückers, Gert Jan Boender, Catherine McCarthy, Dick Heederik, Thomas J. Hagenaars, C. Joris Yzermans, Lidwien A. M. Smit

In S1 File (Table S4), the rows labeled Risk increase and Population Attributable Risk (PAR) in the sheep farms section were swapped. The values currently displayed under Risk increase correspond to PAR, and the values under PAR correspond to Risk increase. It can be viewed below.

## Supporting information

S1 File VGO-UGO supplements. (DOCX)
