# Supplementary material for: Increased risk of pneumonia amongst residents living near goat farms in different livestock-dense regions in the Netherlands
Source: PLoS One. 2023 Jul 5;18(7):e0286972. doi: 10.1371/journal.pone.0286972 (PMC10321607; doi:10.1371/journal.pone.0286972)
Supplement: S1 File — (DOCX) [file pone.0286972.s001.docx]

Supplements accompanying manuscript

Increased Risk for Pneumonia among Residents Living near Goat Farms in different livestock-dense regions in the Netherlands

Aniek Lotterman, Christos Baliatsas, Myrna M.T. de Rooij, Anke Huss, José Jacobs, Michel Dückers, Gert Jan Boender, Catherine McCarthy, Dick Heederik, Thomas J. Hagenaars, C. Joris Yzermans, Lidwien A.M. Smit.

# Tabel S.1

Descriptive; Number and percentage of persons in study population with a livestock farm within a certain radius around their residential address

|  | UGO  (n=65.251) |  |
| --- | --- | --- |
|  | **Number** | **%** |
| Poultry (all) |  |  |
| >2000m | 18.537 | 28,4 |
| 1000-2000m | 26.138 | 40,1 |
| 500-1000m | 14.560 | 22,3 |
| <500m | 6.016 | 9,2 |
| Laying hens/ parental line |  |  |
| >2000m | 30.408 | 46,6 |
| 1000-2000m | 18.110 | 27,8 |
| 500-1000m | 11.439 | 17,5 |
| <500m | 5.294 | 8,1 |
| Broilers |  |  |
| >2000m | 50.849 | 77,9 |
| 1000-2000m | 10.640 | 16,3 |
| 500-1000m | 2.650 | 4,1 |
| <500m | 1.112 | 1,7 |
| Pig farms |  |  |
| >2000m | 9.429 | 14,5 |
| 1000-2000m | 25.347 | 38,8 |
| 500-1000m | 20.969 | 32,1 |
| <500m | 9.506 | 14,6 |
| cattle farms |  |  |
| >2000m | 425 | 0,7 |
| 1000-2000m | 9.416 | 14,4 |
| 500-1000m | 27.672 | 42,4 |
| <500m | 27.738 | 42,5 |
| sheeP farms |  |  |
| >2000m | 27.387 | 42,0 |
| 1000-2000m | 26.699 | 40,9 |
| 500-1000m | 8.429 | 12,9 |
| <500m | 2.736 | 4,2 |
| Mink farms |  |  |
| >2000m | 61.651 | 94,5 |
| 1000-2000m | 2.378 | 3,6 |
| 500-1000m | 1.002 | 1,5 |
| <500m | 220 | 0,3 |

# Tabel S.2

Meta analysis; Associations between the presence of six types of livestock farming within a radius of 500m, 1000m en 2000m of the residential address and pneumonia (OR (95% BI)). Based on results from meta-analysis consisting of logistic regression of individual GPs for all ages in UGO (n=65.251)

|  | 500m | 1000m | 2000m |
| --- | --- | --- | --- |
| Goat farms |  |  |  |
| Base model^1^ | 1,27 (0,83-1,95) | 1,10 (0,89-1,36) | 0,95 (0,83-1,08) |
| Corrected for five types of livestock farms^2^ | 1,18 (0,76-1,83) | 1,15 (0,94-1,41) | 0,98 (0,85-1,14) |
| Corrected for NO_2_^3^ | 1,28 (0,83-1,97) | 1,10 (0,89-1,36) | 0,93 (0,81-1,07) |
| Corrected for age*age^4^ | 1,26 (0,82-1,95) | 1,12 (0,93-1,37) | 0,93 (0,81-1,06) |
| Poultry farms^2^ | 0,98 (0,82-1,17) | 0,98 (0,88-1,09) | 1,06 (0,93-1,21) |
| Laying hens/ parental line^2^ | 0,97 (0,81-1,18) | 1,01 (0,88-1,16) | 0,96 (0,79-1,16) |
| Broilers^2^ | 1,17 (0,80-1,72) | 0,95 (0,77-1,18) | 0,96 (0,83-1,11) |
| Cattle farms^2^ | 0,92 (0,81-1,04) | 0,91 (0,79-1,05) | ^5^ |
| Pig farms^2^ | 1,07 (0,92-1,25) | 1,03 (0,92-1,15) | 0,98 (0,84-1,15) |
| Sheep farms^2^ | 0,85 (0,68-1,06) | 0,99 (0,87–1,13) | 0,96 (0,85-1,07) |
| Mink farms^2^ | 2,18 (1,13-4,21) | 1,39 (1,01-1,93) | 1,51 (1,15-1,98) |

^1^ Adjusted for age, sex and a poultry farm within 2000m

^2^ Adjusted for age, sex, pig or goat farm within 500m, sheep or mink farm within 1000m and a poultry farm within 2000m

^3^ Adjusted for age, sex, a poultry farm within 2000m and NO_2_.

^4^ Adjusted for age, sex, a poultry farm within 2000m and age*age

^5^ No estimation possible. Only 9 pneumonia patients live outside the 2000m radius from a cattle farm.

# Tabel S.3

Meta-analysis; Associations between the presence of goat farms and poultry farms within a radius of 500m, 1000m en 2000m of the residential address and pneumonia in 2014, 2015, 2016 of 2017 (OR (95%CI)) Meta-analysis of results from logistic regression of individual GPs for all ages Gelderland, Overijssel en Utrecht (n=65.251)

|  | 500m | 1000m | 2000m |
| --- | --- | --- | --- |
| Goat farms |  |  |  |
| 2014 | 1,49 (0,88-2,53) | 1,15 (0,90-1,48) | 1,01 (0,87-1,18) |
| 2015 | 1,37 (0,81-2,32) | 1,18 (0,94-1,46) | 1,03 (0,89-1,19) |
| 2016 | 1,30 (0,85-2,00) | 1,11 (0,89-1,39) | 0,98 (0,86-1,13) |
| 2017 | 1,32 (0,86-2,03) | 1,12 (0,90-1,39) | 0,97 (0,84-1,11) |
| Poultry farms |  |  |  |
| 2014 | 1,03 (0,86-1,23) | 0,96 (0,85-1,07) | 1,05 (0,92-1,21) |
| 2015 | 0,98 (0,83-1,16) | 0,96 (0,86-1,07) | 1,05 (0,92-1,20) |
| 2016 | 0,98 (0,83-1,16) | 0,97 (0,87-1,08) | 1,05 (0,90-1,22) |
| 2017 | 0,98 (0,83-1,15) | 0,97 (0,87-1,08) | 1,03 (0,90-1,18) |

# Tabel S.4

Kernel-analysis; Results for a possible association between pneumonia and presence of goat farms and sheep farms in UGO for 2014-2017

|  | 2014 | 2015 | 2016 | 2017 |
| --- | --- | --- | --- | --- |
| Goat farms |  |  |  |  |
| Radius in km | 2 | NA* | 1 | 1.5 |
| Risk increase (%) | 1.9 | NA* | 36.1 | 12.4 |
| PAR (%) | 0.6 | NA* | 2.6 | 2.6 |
| Sheep farms |  |  |  |  |
| Radius in km | 1.5 | 1.5 | 1.5 | 1.5 |
| Risk increase (%) | 5.3 | 14.1 | 13.3 | 9.0 |
| PAR (%) | 11.7 | 35.8 | 33.8 | 31.7 |

* No significant risk increase for pneumonia related to goat farms in 2015

# Table S.5

Sensitivity analysis; Logistic regression model looking into the potential additional effect of goat numbers on top of the effect of presence of a goat farm

|  | OR*  (per IQR goats) | 95%CI | P-value | OR  (goat farm y/n) |
| --- | --- | --- | --- | --- |
| 500m | 0.67 | 0.33 – 1.22 | 0.237 | 1.38 |
| 1000m | 0.93 | 0.82 – 1.05 | 0.255 | 1.14 |
| 2000m | 0.97 | 0.86 – 1.08 | 0.581 | 0.88 |

* This is the added effect on top of the effect of presence of goat farm (y/n)
* Adjusted for presence of goat farm (y/n), sex, age group, presence of poultry farm within 2000m (y/n)
* according to Huijskens et al. ^26^

The model:
pneumonia (y/n)~ mean centered value for number of goats for buffer + presence of goats (y/n) within buffer + sex + age group + presence of poultry farm within 2000m

# Table S.6

Sensitivity analysis; Logistic regression model including number of goats as a categorical variable

|  | OR | 95%CI | P-value |
| --- | --- | --- | --- |
| 500m |  |  |  |
| no goats (indicator) | . | . | . |
| ≤ median number of goats | 1.64 | 0.86 – 2.81 | 0.098 |
| > median number of goats | 1.21 | 0.57 – 2.22 | 0.582 |
| 1000m |  |  |  |
| no goats (indicator) | . | . | . |
| ≤ median number of goats | 1.14 | 0.85 – 1.50 | 0.361 |
| > median number of goats | 1.14 | 0.85 – 1.50 | 0.359 |
| 2000m |  |  |  |
| no goats (indicator) | . | . | . |
| ≤ median number of goats | 0.94 | 0.82 – 1.07 | 0.327 |
| > median number of goats | 0.82 | 0.71 – 0.96 | 0.012 |

Adjusted for sex, age group, presence of poultry farm within 2000m (y/n)
